# Adaptation of Pelage Color and Pigment Variations in Israeli Subterranean Blind Mole Rats, *Spalax Ehrenbergi*

**DOI:** 10.1371/journal.pone.0069346

**Published:** 2013-07-25

**Authors:** Natarajan Singaravelan, Shmuel Raz, Shay Tzur, Shirli Belifante, Tomas Pavlicek, Avigdor Beiles, Shosuke Ito, Kazumasa Wakamatsu, Eviatar Nevo

**Affiliations:** 1 Institute of Evolution, University of Haifa, Mount Carmel, Haifa, Israel; 2 Bommanampalayam, Coimbatore, Tamil Nadu, India; 3 Department of Chemistry, Fujita Health University School of Health Sciences Toyoake, Aichi, Japan; University of Lausanne, Switzerland

## Abstract

**Background:**

Concealing coloration in rodents is well established. However, only a few studies examined how soil color, pelage color, hair-melanin content, and genetics (i.e., the causal chain) synergize to configure it. This study investigates the causal chain of dorsal coloration in Israeli subterranean blind mole rats, *Spalax ehrenbergi*.

**Methods:**

We examined pelage coloration of 128 adult animals from 11 populations belonging to four species of *Spalax ehrenbergi* superspecies (*Spalax galili, Spalax golani, Spalax carmeli*, and *Spalax judaei*) and the corresponding coloration of soil samples from the collection sites using a digital colorimeter. Additionally, we quantified hair-melanin contents of 67 animals using HPLC and sequenced the *MC1R* gene in 68 individuals from all four mole rat species.

**Results:**

Due to high variability of soil colors, the correlation between soil and pelage color coordinates was weak and significant only between soil hue and pelage lightness. Multiple stepwise forward regression revealed that soil lightness was significantly associated with all pelage color variables. Pelage color lightness among the four species increased with the higher southward aridity in accordance to Gloger's rule (darker in humid habitats and lighter in arid habitats). Darker and lighter pelage colors are associated with darker basalt and terra rossa, and lighter rendzina soils, respectively. Despite soil lightness varying significantly, pelage lightness and eumelanin converged among populations living in similar soil types. Partial sequencing of the *MC1R* gene identified three allelic variants, two of which were predominant in northern species (*S. galili* and *S. golani*), and the third was exclusive to southern species (*S. carmeli* and *S. judaei*), which might have caused the differences found in pheomelanin/eumelanin ratio.

**Conclusion/Significance:**

Darker dorsal pelage in darker basalt and terra rossa soils in the north and lighter pelage in rendzina and loess soils in the south reflect the combined results of crypsis and thermoregulatory function following Gloger's rule.

## Introduction

Mammalian pelage coloration plays an important role in crypsis, intra-specific signaling, thermoregulation, and ultraviolet screening [1]–[4]. Several studies demonstrated a strong positive correlation between rodents' coat color and background color of the environment in which they live, indicating that natural selection is operating [5]–[9]. Such adaptive coat color variations are caused by the switch between ‘brown to black’ eumelanin and ‘yellow to red’ pheomelanin [10]–[13]. This dual melanogenesis is controlled by the interaction of two proteins: melanocortin-1-receptor (MC1R) and agouti-signaling protein (ASIP) [14]–[16]. *MC1R* is a G protein-coupled receptor expressed highly in melanocytes involved in the production of eumelanin. Agouti is an antagonist of *MC1R.* The expression of ASIP suppresses the synthesis of eumelanin and triggers the production of pheomelanin.

A classic example of *MC1R* gene-driven coat color variation is demonstrated in pocket mice; individuals inhabiting dark volcanic lava have dark coats, and mice inhabiting light-colored granitic rocks exhibit light coats. This color polymorphism is considered as a cryptic adaptation to avoid predation [17], [18]. Despite numerous studies on the dorsal coloration of rodents, how melanin contents are selected to form concealing coloration is least explored [19]. A clearer understanding is essential to elucidate the evolution of concealing coloration and the pigmental variation underlying it, including comparisons of: ‘soil color vs. pelage color’, ‘pelage color vs. melanin contents’, and ‘pelage color vs. candidate gene’, to suggest adaptation to soil color. Such comprehensive studies are still lacking. Hence, in the present study, we intend to address the above scenario in populations of the subterranean mole rats of the *Spalax ehrenbergi* superspecies in Israel.

Diversifying selection of pelage color occurs even in burrowing subterranean mammals that exhibit adaptation of pelage to the color of their background habitat (e.g., *Thomomys* [20] and *Geomys* [21]). Such observations were substantiated also in blind mole rats of the *Spalax ehrenbergi* superspecies whose populations live in the subterranean environment and are selected for different soil colors by differential predation (22). The pelage coloration of *Spalax ehrenbergi* varies and tends to match different soil colors [22]. Why and how selection works on the pelage color of these blind mole rats, restricted most of their lives to the underground ecotope, is important evolutionarily. There are four species of mole rats of the superspecies “*Spalax ehrenbergi*” in Israel; *S. galili* (2*n* = 52), *S. golani* (2*n* = 54), *S. carmeli* (2*n* = 58), and *S. judaei* (2*n* = 60), whose distribution in Israel in four distinct parapatric, climatically different areas is highly correlated with increasing aridity, both southward and eastward [23]–[25].

Pelage of blind mole rats is usually gray, but there are differences among the four species which enable them to be camouflaged above ground, especially at night [22]. The apical and sub-apical portion of the hairs of the northern mesic-species (2*n* = 52, 54) is reddish orange with darker pelage corresponding to reddish brown and dark tones of the terra rossa and basalt soils, respectively, which they mainly inhabit. The xeric southern species (2*n* = 58, and primarily 2*n* = 60) tend to be yellowish with lighter pelage, except for those populations living in the alluvial soils around the coastal rivers. Such color variation between species of mole rats is hypothesized to be due to the selection pressures of predation and thermoregulation, even though they spend little time above ground (mostly at night). The correlation of pelage color with soil color certainly suggests that adaptation occurs and reflects an underlying genotypic variation. The study by Heth et al. [22] relied on Munsell color charts to determine both pelage and soil colors. This method is limited due to eye perception differences, illumination, and other factors that can affect color determination. In the current study, we measure the coloration of mole rats using a digital colorimeter. We test the working hypothesis that crypsis exists in mole rats when there is harmony in the causal chain (i.e., soil color, pelage color, hair melanin contents, and genetics) of pelage coloration. Complementarily, we explore the climatic cline on pelage color following the ecological Gloger's rule.

## Methods

### Ethics statement

Mole rats were trapped from various locations using special live traps by exposing the tunnels using a hoe. No specific permissions were required for these locations, which are wild habitats of mole rats; none belonged to protected areas or belonged to private property. The field studies did not involve endangered or protected species. Animals used in the study were adults. The experiments were approved by the Ethics Committee of the University of Haifa.

### Animal collection and hair sampling

**Figure 1:**
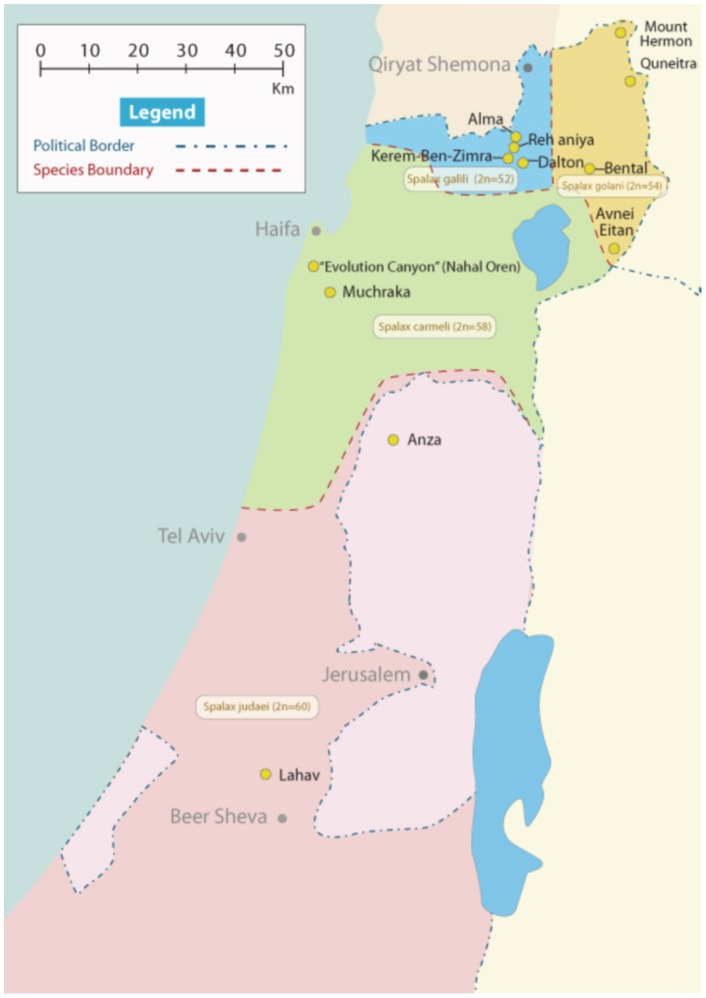
Map shows the studied populations of mole rats in Israel. Kerem-Ben-Zimra, Alma and Rehaniya populations are located in Galilee mountains; Quneitra¸ A. Etan and Bental are in Golan heights. Muhraka and “Evolution Canyon” (Nahal Oren) populations are in Carmel mountains. Anza is located in West Bank, while Lahav is in Negev.

We measured the dorsal pelage color of 128 adult mole rats from 11 populations of the four *Spalax* species in Israel (Figure 1). Mole rats were captured in the field during 2002–2010 and were maintained in the animal facility at the Institute of Evolution, University of Haifa, under constant conditions (22°C with relative humidity 70%; photoperiod 12L:12D) and were fed vegetables. Hair samples were excised with scissors to the full length obtained from random locations over the animal's dorsal body across a 3×3 cm^2^ area (∼60 mg of hairs) from the different geographic regions. We made sure to excise uniform lengths (∼95% of total length) of the pelage. This procedure was consistent for all individuals to standardize sample collections for melanin analysis. Animals were treated with care while removing hairs to prevent injury and/or suffering.

### Color Measurement

All color measurements were made on adults. Note that the coloration changes during the course of ageing; pups and juveniles are lighter than fully matured adults. We measured the dorsal pelage color using a digital colorimeter (Spec boss 4000, JETI). Each animal was measured at least 12 times in random locations over the dorsal body. We used the L*a*b* color space model (CIE-LAB) under standard daylight illumination [26] to quantify different components of the measured color. This color space was selected because it is more appropriate for the biological aims of this study. The color space component ‘L*’ represents the level of lightness in color (L* estimates equivalent to ‘brown to black’ eumelanin), a positive value of ‘a*’ is represented in red/magenta, while a negative value of ‘a*’ is represented in green. The positive value of ‘b*’ is represented by the amount of purplish-red (magenta) yellow, while the negative value of ‘b*’ represents blue (a* and b* estimates are equivalent to the ‘yellow to red’ pheomelanin). Chroma (‘C’ =  √a*^2^+b*^2^) and hue (‘h’ =  arc tan [a*/b*]) were derived from L*a*b* parameters and were also included in the analysis. For statistical analysis, we estimated the average value for each color component, for each individual, following elimination of the 20% percentile of the lowest and highest color measurements in order to avoid the inclusion of unreliable estimates.

### Melanin Assay

Micro-analytical methods to quantify the amounts of eumelanin and pheomelanin were based on the formation of specific degradation products, pyrrole-2,3,5-tricarboxylic acid (PTCA) by alkaline H_2_O_2_ oxidation of eumelanin and 4-amino-3-hydroxyphenylalanine (4-AHP) by reductive hydrolysis of pheomelanin with hydriodic acid (HI) [27], [28]. Hair samples were homogenized with a Ten-Broeck glass homogenizer at a concentration of 10 mg/mL water.

Alkaline H_2_O_2_ oxidation was used to measure eumelanin (PTCA). A sample homogenate (100 µL) was taken in a 10-ml screw-capped conical test tube, to which 375 µL 1 mol/L K_2_CO_3_ and 25 µL 30% H_2_O_2_ (final concentration: 1.5%) were added. The mixture was mixed vigorously at 25°C±1°C for 20 h. The residual H_2_O_2_ was decomposed by adding 50 µL 10% Na_2_SO_3_, and the mixture was then acidified with 140 µL 6 mol/L HCl (the generation of CO_2_ occurs by adding HCl to the alkaline mixtures). After vortex-mixing, the reaction mixture was centrifuged at 4,000 g for 1 min, and an aliquot (80 µL) of the supernatant was directly injected into the HPLC system [28]–[30].

HI reductive hydrolysis was used to measure pheomelanin (4-AHP). A sample homogenate (100 µL) was taken in a 10-ml screw-capped conical test tube to which 20 µL 50% H_3_PO_2_ and 500 µL 57% HI were added. The tube was heated at 130°C for 20 h, after which the mixture was cooled. An aliquot (100 µL) of each hydrolysate was transferred to a test tube and evaporated until dried using a vacuum pump connected to a dry ice-cooled vacuum trap and two filter flasks containing NaOH pellets. The residue was dissolved in 200 µL 0.1 mol/L HCl. An aliquot (10 µL) of each solution was analyzed on the HPLC system [27].

Soluene-350 solubilization was used to measure total melanin. A sample homogenate (100 µL) was taken in a 10-ml screw-capped conical test tube, to which 900 µL Soluene-350 (from Perkin-Elmer) was added. The tube was vortex-mixed and heated at 100°C (in a boiling water bath) for 15 min, after which the mixture was cooled. The tube was vortex-mixed and heated again at 100°C for an additional 15 min and then cooled. After vortex-mixing, the mixture was centrifuged at 4,000 g for 3 min, and the supernatant was analyzed for absorbance at 500 nm (A500). For a reference, a mixture of 100 µL water and 900 µL Soluene-350 was used after heating under the same conditions as for the samples. Background values (due to protein) of 0.019 at 500 nm and 0.001 at 650 nm for mouse hair samples were recorded [31].

### HPLC analyses

H_2_O_2_ oxidation products were analyzed with the HPLC system consisting of a JASCO 880-PU liquid chromatograph (JASCO Co., Tokyo, Japan), a Shiseido C_18_ column (Shiseido Capcell Pak MG; 4.6×250 mm; 5 µm particle size), and a JASCO UV detector. The mobile phase was 0.1 mol/L potassium phosphate buffer (pH 2.1): methanol, 99: 1 (v/v). Analyses were performed at 45°C at a flow rate of 0.7 mL/min. Absorbance of the elute was monitored at 269 nm. A standard solution (80 µL) containing 1 µg each of PTCA (pyrrole-2,3,5-tricarboxylic acid), PDCA (pyrrole-2,3-dicarboxylic acid), TTCA (thiazole-2,4,5-tricarboxylic acid), and TDCA (thiazole-2,3-dicarboxylic acid) in 1 mL HPLC buffer was injected into the HPLC system every 10 samples.

HI reductive hydrolysis products were analyzed with an HPLC system consisting of a JASCO 880-PU liquid chromatograph, a JASCO C_18_ column (JASCO Catecholpak; 4.6×150 mm; 7 µm particle size), and an EICOM ECD-300 electrochemical detector. The mobile phase used for analysis of 4-AHP was 0.1 mol/L sodium citrate buffer, pH 3.0, containing 1 mmol/L sodium octanesulfonate and 0.1 mmol/L Na_2_EDTA: methanol, 98: 2 (v/v). Analyses were performed at 35°C at a flow rate of 0.7 mL/min. The electrochemical detector was set at +500 mV versus an Ag/AgCl reference electrode. A standard solution (10 µL) containing 500 ng each of 4-AHP (4-amino-3-hydroxyphenylalanine) and 3-AHP (3-amino-4-hydroxyphenylalanine; 3-aminotyrosine from Sigma) in 1 mL 0.1 mol/L HCl was injected into the HPLC system every 10 samples.

### Screening variations in the *MC1R* gene of mole rats

The *MC1R* gene was partially sequenced (905 bp out of 952 bp total length) in 68 mole rats from the four *Spalax* species, as presented in Supplemental Table S1. Specific PCR primers were used to amplify the gene fragment: S5`F ‘cagaagaggctgctggactc’ and S3`B ‘gagctccgcatgacactcag’. PCR was performed in a 25-µL reaction volume containing 12.5 µL ReadyMixTM Taq PCR Reaction Mix with MgCl2 (Sigmàs product code P4600), 0.5 µL primer s5f 0.5 µL primer s3b, 10.5 µL water, and 1 µL DNA sample. PCR-amplification was done under the following conditions: 94°C for 5 min, then 39 cycles of 94°C for 30 s, 57°C for 30 s, and 72°C for 30 s, followed by 72°C for 7 min.

### Statistical analysis

Numerical data were shown as means ± SD (standard deviation). The differences in pelage color coordinates and hair melanin contents between males and females, and between populations (where applicable) were examined using the Mann-Whitney test. The Kruskal Wallis test was employed to detect the significance of variation in soil and pelage color coordinates and in hair-melanin contents among populations. The association between soil and pelage color coordinates was detected by employing the Spearman correlation. We used multiple-regression, followed by stepwise forward-regression to reveal the effect of soil color variables (as independent variables) on pelage color variables (as dependent variables) of mole rats. We employed the chi-square test to detect variations in haplotype frequencies between northern and southern species of mole rats.

## Results

### Soil color variations

The soil colors of different populations were light gray, yellow, brown, and dark gray. *S. galili* inhabits a variety of soils including terra rossa, rendzina, and basalt. *S. golani* inhabits basalt soil, *S. carmeli* inhabits terra rossa, and *S. judaei* inhabits rendzina and loess soils. Basalt and terra rossa soils are dark due to the presence of oxide metals, while rendzina and loess soils are light due to the high content of chalk. These variations in soil types are clearly manifested in the measured values of soil color. The maximum lightness score (‘L*’) of soils in our samples is found in *S. judaei* (range L*  = 41.93–45.19) and *S. galili* in the Kerem Ben Zimra rendzina sample we tested (42.19–50.81), compared with *S. carmeli* (23.13–30.93) and *S. golani* (26.36–30.30). In addition, soils of *S. galili* (a* =  −0.84–10.47) and *S. golani* (a* =  5.16–8.08) tend to be more reddish, while *S. judaei* (b* = 13.58–15.94) and *S. galili* (b* =  9.32–16.56) tend to be more yellowish in our samples. As expected, basalt and terra rossa soils exhibited lower lightness, and rendzina and loess soils exhibited higher lightness scores indicating that the former two soil types are relatively darker than the latter, as is also clearly observed. Moreover, terra rossa soils are more reddish, whereas rendzina soils are more yellowish (Table 1). The rendzina soil in Kerem Ben Zimra (L* = 46.62±2.59) is the lightest, and the abutting basalt soil in Alma (26.31±5.73) is the darkest among populations investigated. Rihaniya soil is lighter than the Muhraka and “Evolution Canyon” (Nahal Oren) populations inhabiting terra rossa soils. Soil color parameters varied significantly among populations that live in similar soil types (see Table 1).

**Table 1:** Soil color variation among populations living in different soil types.

Soil**Type	Population (Species)	*N*	L*	a*	b*	C	h
Basalt	Alma (*Spalax galili*)	7	26.31±5.73	8.62±4.18	11.14±0.50	14.57±1.17	53.84±17.72
Basalt	Dalton (*Spalax galili*)	6	30.59±0.94	6.23±0.43	9.97±0.43	11.76±0.59	58.01±0.81
Basalt	Quneitra (*Spalax golani*)	7	27.20±0.57	5.68±0.27	10.39±0.86	11.84±0.87	61.27±1.20
	*Kruskal-Wallis*		*H = 13.972, P<0.001*	*H = 8.764, P = 0.013*	*H = 11.599, P = 0.003*	*H = 9.779, P = 0.008*	*H = 10.533, P = 0.005*
Terra rossa	Rihaniya (*Spalax galili*)	8	28.97±0.82	10.07±0.29	12.77±0.34	16.26±0.42	51.74±0.57
Terra rossa	Muhraka (*Spalax carmeli*)	9	28.89±1.41	7.32±0. 63	11.08±0.87	13.29±0.93	56.51±2.32
Terra rossa	“Evolution Canyon” Nahal**Oren (*Spalax carmeli*)	8	26.32±2.13	3.31±0.66	6.78±0.96	7.55±1.12	64.22±0.85
	*Kruskal-Wallis*		*H = 9.109, P = 0.011*	*H = 21.350, P = <0.001*	*H = 21.040, P = <0.001*	*H = 21.350, P = <0.001*	*H = 20.739, P = <0.001*
Rendzina	Kerem-Ben-Zimra (*Spalax galili*)	14	46.62±2.59	5.78±0.11	15.95±0.37	16.969±0.378	70.04±0.186
Loess	Lahav (*Spalax judaei*)	7	43.32±1.33	5.23±0.25	14.75±1.04	15.65±1.06	70.46±0.430

Footnote: ‘L*’ represents the level of lightness of the color, positive value of ‘a*’ represents red. Value of ‘b*’ represents the amount of purplish -red (magenta). Chroma (‘C’ =  √a*^2^+b*^2^) and hue (‘h’ =  arc tan [a*/b*]) were calculated from L*a*b* parameters.

### Variation between sexes

We did not find differences in pelage color between females and males (species and populations pooled data) both in the colorimeter measurements that were performed on 30 females and 13 males (Table 2), and in the comparison of the melanin content, which were performed on 47 females and 20 males (Table 3). These results firmly support the conclusion that mole rats do not show sex color variation in pelage color as expected from blind species.

**Table 2:** Pelage color variation between sexes (species and populations' pooled data).

Sex	*N*	L*	a*	b*	C	h
Female	30	37.60±2.53	0.80±0.53	4.25±1.52	4.34±1.58	81.45±5.74
Male	13	37.38±2.43	0.77±0.42	3.49 ±1.15	3.60±1.18	80.44 ±9.65
*Mann-Whitney*		*U = 196.00; P = 0.989*	*U = 189.00; P = 0.884*	*U = 258.00; P = 0.098*	*U = 255.00; P = 0.116*	*U = 232.00; P = 0.334*

**Table 3:** Hair-melanin content variation between sexes (species & populations' pooled data).

Sex	*N*	PTCA	4-AHP	TM
Female	47	2038±452	49±31	0.76±0.19
Male	20	1949±514	64±44	0.66±0.13
*Mann-Whitney*		*U = 506.50; P = 0.622*	*U = 384.50; P = 0.244*	*U = 622.00; P = 0.038*

### Variation among populations in pelage color

Overall, pelage lightness (L*) varied significantly among 11 populations from the four species living in different soils (*H = 20.366, df = 10, P = 0.026*). Likewise, pelage color lightness of populations living in four soil types (basalt, terra rossa, rendzina, and loess) varied significantly (*H = 10.399, df = 3, P = 0.015*; populations' pooled data). Regardless of species and climatic divergence, the Anza population of *S. judaei* (39.89±3.63) from the south and Kerem-Ben-Zimra, KBZ population of *S. galili* (38.66±2.66) from the north, living 70 km apart, were the lightest, both inhabiting rendzina (light colored, chalky) soils. The KBZ population inhabits a significantly more humid region. The Rihaniya population of *S. galili* that lives in the darker terra rossa soil, 2 km apart from KBZ, was the darkest (32.32±8.50) (Table 4). This indicates how cryptic factors prevail over climate factors in nearby populations. Among basalt populations, Alma (38.11±4.18) exhibited the lightest pelage color and neighboring Dalton (34.49±6.38) exhibited the darkest. The Anza population showed lighter pelage than the Kerem-Ben-Zimra in the light rendzina soil, which may indicate the climatic determinant in Anza, which is drier than KBZ. Among populations that live in terra rossa soil, mole rats in “Evolution Canyon”, Nahal Oren (38.07±3.50) exhibited the lightest pelage, and those living in Rihaniya (32.32±8.50) showed the darkest pelage (Table 4). Regardless of species, populations living in similar soil types did not vary significantly in lightness of pelage color (Table 4). However, populations living on basalt showed significant variations in color coordinates, a* (*H = 33.617, df = 4, P<0.001*), b* (*H = 29.466, df = 4, P<0.001*), chroma (*H = 30.521, df = 4, P<0.001*), and hue (*H = 17.739, df = 4, P = 0.001*). Populations that live in terra rossa soils exhibited significant variations only for pelage variables a* (*H = 12.974, df = 2, P = 0.002*) and h* (*H = 15.384, df = 2, P<0.001*); the rendzina populations did not show significant variation for any of the pelage color variables (Table 4). Population-wise variations are striking across 11 populations living in four soil types. But regardless of species, populations living in similar soil types showed convergence in pelage lightness (Table 4), again indicating the soil determinant as an important variable in determining pelage color.

**Table 4:** Pelage color variation among populations living in different soil types.

Soil**Type	Population (Species)	*N*	L*	a*	b*	C	h
Basalt	Alma (*Spalax galili*)	21	38.11±4.18	2.20±1.02	6.68±2.29	7.05±2.47	72.14±4.60
Basalt	Dalton (*Spalax galili*)	13	34.49±6.38	2.01± 0.64	6.50±1.92	6.82±1.99	72.10±3.96
Basalt	Quneitra (*Spalax golani*)	15	35.91±2.06	0.67±0.33	2.94±1.03	3.04±1.06	80.50±9.46
Basalt	A. Etan (*Spalax golani*)	4	36.79±2.49	1.36±0.50	5.46±1.30	5.64±1.36	76.95±3.61
Basalt	Bental (*Spalax golani*)	5	36.89±3.25	1.10±0.32	4.59±1.04	4.72±1.06	77.08±3.00
	*Kruskal-Wallis*		*H = 6.164, P = 0.187*	*H = 33.617, P<0.001*	*H = 29.466, P<0.001*	*H = 30.521, P<0.001*	*H = 17.739, P = 0.001*
Rendzina	Kerem-Ben-Zimra (*Spalax galili*)	20	38.66±2.66	1.96±1.38	6.24±2.54	6.58±2.81	75.62±7.30
Rendzina	Anza (*Spalax judaei*)	13	39.89±3.63	1.56±1.02	5.74±2.17	5.99±2.31	79.31±12.23
	*Mann-Whitney*		*U = 97, P = 0.231*	*U = 142, P = 0.672*	*U = 142,P = 0.672*	*U = 143, P = 0.645*	*U = 129, P = 0.985*
Terra rossa	Rihaniya (*Spalax galili*)	8	32.32±8.50	1.90±0.90	5.86±2.78	6.18±2.90	71.06±5.18
Terra rossa	Muhraka (*Spalax carmeli*)	13	36.69 ±2.02	0.46±0.26	3.55±1.08	3.59±1.09	85.62±5.52
Terra rossa	“Evolution Canyon” Nahal**Oren (*Spalax carmeli*)	8	38.07±3.50	0.90±0.92	4.31±2.02	4.44±2.15	82.95±9.77
	*Kruskal-Wallis*		*H = 2.827, P = 0.243*	*H = 12.974, P = 0.002*	*H = 4.978, P = 0.083*	*H = 5.798, P = 0.055*	*H = 15.384, P<0.001*
Loess	Lahav (*Spalax judaei*)	8	37.80±3.32	1.17±0.54	4.84±1.37	5.00±1.44	78.51±3.92

### Hair-melanin contents

Similar to the measured pelage color, PTCA (eumelanin) contents of mole rat populations living in similar soil types did not vary in all three soil types (see Table 5): basalt (*U* = 54.00; *P* = 0.791), rendzina (*U* = 33.00; *P* = 0.212), and terra rossa (*U* = 47.5; *P* = 0.672). However, 4-AHP (pheomelanin) contents did vary among populations of mole rats living in rendzina (*U* = 7.00; *P* = 0.001) and terra rossa (*U* = 18.0; *P* = 0.047) but did not vary in basalt (*U* = 116.00; *P* = 0.427).

**Table 5:** Hair-melanin content variation among populations living in different soil types.

Soil Type	Population (Species)	*N*	PTCA	4-AHP	TM
Basalt	Alma (*Spalax galili*)	10	2050±584	74±48	0.57±0.14
Basalt	Quneitra (*Spalax golani*)	10	2040±500	60±48	0.67±0.12
	*Mann-Whitney*		*U = 54.00; P = 0.791*	*U = 116.00; P = 0.427*	*U = 81.00; P = 0.076*
Rendzina	Kerem-Ben-Zimra (*Spalax galili*)	10	1873±459	76±35	0.63±0.16
Rendzina	Anza (*Spalax judaei*)	10	1641±371	29±17	0.68±0.14
	*Mann-Whitney*		*U = 33.00; P = 0.212*	*U = 7.00; P = 0.001*	*U = 61.00; P = 0.427*
Terra rossa	Muhraka (*Spalax carmeli*)	12	2201±439	32±10	0.88±0.20
Terra rossa	“Evolution Canyon” Nahal Oren (*Spalax carmeli*)	7	2132±251	53±28	0.87±0.11
	*Mann-Whitney*		*U = 47.5; P = 0.672*	*U = 18.0; P = 0.047*	*U = 42.00; P = 0.966*
Loess	Lahav (*Spalax judaei*)	8	2178±423	55±22	0.82±0.08

### Pelage color vs soil color

The lightness of *Spalax* pelage is significantly correlated with the soils' hue (Spearman's r = 0.617, P = 0.043, N = 11). Multiple forward stepwise regression between soil color variables (as independent variables) and pelage color coordinates (as dependent variables) revealed that soil lightness (L*) influenced all pelage color variables (Table 6). The combined (i.e., average) R (0.4) of the multiple regressions signifies low to intermediate regression; yet pelage L* and h* are partially influenced by soil color (as the constant value is large in the equation: 34.42 for L* and 68.59 for h*). Populations living in darker basalt and terra rossa soils exhibited darker pelage, and lighter rendzina and loess soils exhibited lighter pelage. Among the four soil types in which the mole rats were studied, rendzina soil has significant impact on pelage coloration, which revealed similarities in scores of all color variables between populations of *S. galili* and *S. judaei* (Table 4), despite being the distant populations (Figure 1), separated by dozens of kilometers. By contrast, abutting populations living on drastically different soil types (such as KBZ on rendzina and Dalton on basalt) differ in pelage color exemplifying cryptic coloration.

**Table 6:** Association between soil color on pelage color of mole rats (forward stepwise multiple regressions).

Dependent variables	Step	Independent determinants	R	R^2^	F	*P*
Pelage L*	0 (forced)	soil L*	0.099			
Pelage L*	1	soil L*, soil b*	0.277	0.077	4.649	0.011
Pelage a*	0 (forced)	soil a*	0.155			
Pelage a*	1	soil a*, L*	0.352	0.124	7.908	<0.001
Pelage a*	2	soil a*, L*, C*	0.495	0.245	12.033	<0.001
Pelage b*	0 (forced)	soil b*	0.091			
Pelage b*	1	soil b*, C*	0.283	0.081	4.893	0.009
Pelage b*	2	soil b*, C*, L*	0.476	0.226	10.815	<0.001
Pelage b*	3	soil b*, C*, L*, a*	0.492	0.242	8.793	<0.001
Pelage b*	4	soil b*, C*, L*, a*, h*	0.496	0.24	7.105	<0.001
Pelage C	0 (forced)	soil C*	0.186			
Pelage C	1	soil C*, b*	0.289	0.084	5.109	0.008
Pelage C	2	soil C*, b*, L*	0.481	0.231	11.111	<0.001
Pelage C	3	soil C*, b*, L*, a*	0.497	0.247	9.011	<0.001
Pelage C	4	soil C*, b*, L*, a*, h*	0.500	0.250	7.267	<0.001
Pelage h	0 (forced)	soil h*	0.220			
Pelage h	1	soil h*, L*	0.395	0.156	10.330	<0.001
Pelage h	2	soil h*, L*, b*	0.399	0.159	7.013	<0.001
Pelage h	3	soil h*, L*, b*, a*	0.420	0.176	5.880	<0.001
Pelage h	4	soil h*, L*, b*, a*, C*	0.428	0.183	5.891	<0.001

Footnote: ‘L*’ represents the level of lightness of the color, positive value of ‘a*’ represents red. Value of ‘b*’ represents the amount of purplish-red (magenta). Dependent variables were the pelage color coordinates of mole rats and the independent variables consisted of soil color coordinates. Criterion for the variable to enter the regression F>0.300, to be removed F<0.100.

# The program calculates the coefficient of the forced variable in the equation, and it is entered as R.

## The F and P are calculated by ANOVA.

### Hair-Melanin Content vs. Pelage and Soil Colors

Hair-melanin contents in the pelage of mole rats reflected the pelage coloration trend. As shown by pelage color scores (Table 4), populations inhabiting darker soils, like basalt or terra rossa, exhibited higher PTCA (eumelanin) contents; basalt-inhabiting populations, such as Alma (2050±584 ng/mg) and Quneitra (2040±500 ng/mg), and terra rossa-inhabiting populations, like Muhraka (2201±439 ng/mg) and N. Oren (2132±251 ng/mg), had more PTCA than the lighter rendzina populations [Kerem-Ben-Zimra (1873±459) and Anza (1641± 371) populations (see Table 5)]. Likewise, 4-AHP (pheomelanin) is higher in the hairs of mole rat populations with higher pelage scores of a* (red magenta) and b* (magenta/yellow) – variables, which are likely to determine pheomelanin [e.g., Alma (74±48 ng/mg) and Kerem-Ben-Zimra (76±35 ng/mg)], whereas it is lower in populations with lower pelage scores a* and b* [e.g., Muhraka (32±10) and “Evolution Canyon”, Nahal Oren (53±28)] (see Tables 4 and 5). Populations with higher reddish and yellowish shades to their pelage exhibit higher amounts of pheomelanin contents in their hairs.

Similarly, hair-melanin content appeared to be associated with soil color as well; for example, populations inhabiting darker soil (e.g., Alma, Quneitra and “Evolution Canyon” Nahal Oren) exhibited higher eumelanin contents, and populations inhabiting darker basalt soils (Alma and Quneitra), with higher scores of a* and b*, exhibited higher pheomelanin and lower scores of a* and b* in pelage in “Evolution Canyon” (Nahal Oren) populations led to lower pheomelanin content (see Tables 1 and 5).

### Variations in the *MC1R* gene of mole rats

**Figure 2:**
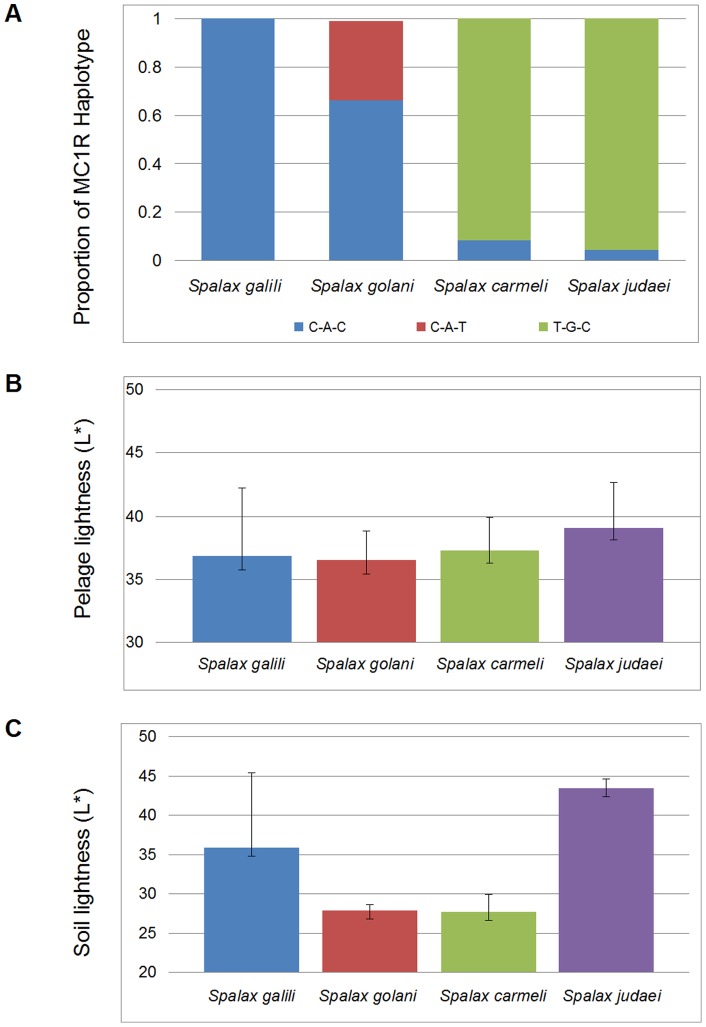
Patterns of variation across species of mole rats in Israel. A) genetic variation, B) phenotypic variation, and C) environmental variation.

**Figure 3:**
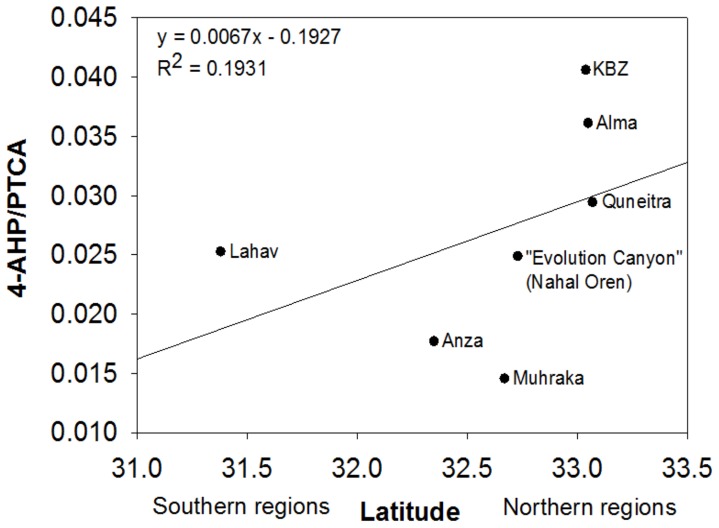
Pheomelanin/eumelanin ratio among *Spalax* populations across latitude.

The *MC1R* gene was sequenced in 68 samples. The sequences were compared to the consensus reference sequence. We identified three variants in this sample set: 1) a synonymous substitution in position c.228 C to T; 2) a non-synonymous substitution in position c.502 A to G, which changes the amino acid from methionine to valine (c.502A>G; p.168 M>V); and 3) a synonymous substitution in position c.592 C to T. The first and second mutations were in high linkage disequilibrium in all of the tested animals. We found three haplotypes: C-A-T (the consensus sequence), C-A-C, and T-G-C. C-A-C and C-A-T are almost restricted to northern species (*S. galili* and *S. golani*, see Figure 2A–C & Table S2); while C-A-C haplotype occurs in each of the southern species (*S. carmeli* and *S. judaei*), the latter is exclusive to *S. golani* (Supplemental Table S2), whereas T-G-C is restricted to southern species (*S. carmeli* and *S. judaei,* see Figure 2C and Supplemental Table S2). Thus, there is a clear separation between the northern (*S. galili* and *S. golani*) and southern species (*S. carmeli* and *S. judaei*); χ^2^(2)  = 60.5, *p*<0.001. Notably, northern populations with C-A-C haplotype exhibited significantly higher pheomelanin than southern populations with T-G-C haplotype (U = 318.5, N_1_ = 30, N_2_ = 37; P = 0.003), whereas eumelanin did not vary very much (U = 605, N_1_ = 30, N_2_ = 37; P = 0.533). Conceivably, the pheomelanin/eumelanin ratio of populations increased northward (Figure 3). But, the pelage lightness estimator exhibited a linear trend, and the association between *MC1R* haplotypes and pelage lightness (among populations) remains unclear. Nevertheless, the *MC1R* haplotypes completely diverged between the northern and the southern species of mole rats (Figure 2A–C), in correlation with pheomelanin concentrations, suggesting that it is subjected to climatic selection.

## Discussion

### Overview

Neither pelage coloration nor hair-melanin contents of mole rats vary between the visually identical males and females, and this confirms there is no sexual dichromatism that is conceivable, as *Spalax* is a completely blind mammal. Habitat soil coloration, pelage coloration, and hair-melanin contents varied among the populations. Soil lightness (L*) was a determinant of all pelage color variables. The pelage lightness increased with increasing aridity regionally across Israel. Among mesic north populations, KBZ displayed lighter pelage on lighter rendzina soil. How far do these results corroborate with the three functions of coat coloration: intraspecific communication, crypsis, and thermoregulation [8]? Intraspecific communication cannot be the case with *Spalax ehrenbergi* superspecies as the animal cannot use apparent coloration-based visual cues. Therefore, blending with the background environment to evade easy detection by predators and thermoregulation could be the only reasons behind coat color variation in mole rats.

### Does pelage coloration exemplify ‘crypsis’?

#### Driving forces – soil color or soil type?

Darker basalt and terra rossa soils selected for darker pelage, whereas lighter rendzina selected for lighter pelage despite variation between populations and species. Pelage colors of populations living in heterogeneous soils varied; yet pelage lightness and eumelanin contents in the hairs of mole rat populations living in similar soil type did not vary much, despite soil lightness varying among these populations (Table 4). Though soil color is a strong evolutionary force driving pelage coloration [32], [33], the soil type overrides soil color and selects for convergent evolution in pelage lightness and eumelanin of the *Spalax ehrenbergi* superspecies. These results support Hardy's [34] findings that soil types have significant influence on coat color and on the local distribution of mammals. Indeed pelage coloration of different populations approximated better with soil type than with microscale variation in soil color. Populations that inhabit lighter rendzina (Kerem-Ben-Zimra and Anza) and loess (Lahav) soils exhibited lighter pelage, and those that inhabit darker basalt (Dalton, Quneitra, A. Etan, and Bental) and terra rossa soils (Rihaniya and Muhraka) exhibited darker pelage (Table 4). Similarly, the populations inhabiting darker basalt (e.g., Alma, Quneitra) and terra rossa (e.g., Muhraka and “Evolution Canyon”, Nahal Oren) soils exhibited higher amounts of PTCA (eumelanin) contents than populations living in lighter rendzina (Kerem-Ben-Zimra and Anza) that showed lower eumelanin contents (Table 5). Alma and Kerem-Ben-Zimra populations showed higher a* and b* scores (which would likely determine the prevalence of pheomelanin contents in pelage) in soil, resulting in higher pheomelanin contents (see Tables 1 & 5). Altogether, the results indicate that both coloration and melanin contents of pelage are in accordance with soil type. The role of abrasive properties of the three soil types on both eumelanin and pheomelanin in the hairs (or pigment-type switching) is expected, but remains to be investigated.

In essence, the pelage lightness of mole rats show macro-geographic variations, but on a microscale, only the KBZ population exhibited lighter pelage on lighter soil color. Thus, the selection on pelage coloration towards crypsis is weak, but certainly exists. Nevertheless, the pelage coloration of a sympatric species, such as the spiny mouse *Acomys cahirinus*, in “Evolution Canyon” (Nahal Oren) responded well to variations in soil color even on a microscale [19]. This suggests that selection for crypsis might be stronger in such aboveground rodents than in subterranean mole rats, as the predation pressure on the former and latter varies drastically. Perhaps, aboveground vegetation, texture, and microclimatic differences among habitats might lead to the weak correlations between soil and pelage in Israeli mole rats (see also Rios et al. [35]).

### Pelage coloration for thermoregulation

#### Climatic cline

Mole rats live in underground burrows that are microclimatically more or less stable [36]. However, soil moisture and temperature vary in their burrows across the climatically-divergent regions during seasonal changes [37]. Furthermore, mole rats seldom spend time aboveground during day time. Both soil moisture and aboveground activity together might influence the pelage coloration to regulate thermoregulation. Mole rats in Israel were distributed across a gradient of increasing aridity southward along the ranges of 2*n* = 52 → 2*n* = 58 → 2*n* = 60 and eastward (2*n* = 52→54) [24]. *S. carmeli* and *S. judaei* inhabit drier environments; therefore, their pelage is lighter. This could be in part to offset the hotter climate [22], in addition to its possible role in concealing coloration.

Though we don't have direct field-based evidence on whether *Spalax ehrenbergi* regulates body temperature during different seasons, a laboratory study by Haim et al. [38] shows that they might. Exposing cold-sensitive individuals to short photoperiods (8L:16D) at an ambient temperature (T_a_) of 22°C, increased their thermoregulatory capacity under cold conditions (6°C for 6 h) when compared to individuals that were acclimated to 12L:12D at the same T_a_. Conversely, acclimation of cold-resistant individuals to T_a_  = 17°C, but with a photoperiod of 16L:8D, decreased thermoregulatory capacity. It has been postulated that blind mole rats detect changes in photoperiods through its atrophied eyes or by other means involving the melatonin pathway [38]. Yet, how far mole rats could differentiate the already limited photic cues inside its burrow and then across seasons needs further research.

### Soil and Climate – The dual factor

The coat coloration in rodents correlates both with the habitat gradients and climatic clines [39]. Animals in humid environments tend to be dark, whereas conspecifics in arid environments are light, a generalization recognized as Gloger's rule [40]. The pelage lightness (L*) score across four *Spalax* species in Israel obeys this rule; *S. galili* and *S. golani* are darker and live relatively in humid environments, and *S. carmeli* and *S. judaei* (particularly the latter) are lighter and live in arid environments. Based on the comparisons of climatic data presented in Auffray et al. [41] and color scores of different variables in both Heth et al. [22] and the present study, some high-humid populations (e.g., KBZ population of *S. galili* and “Evolution Canyon”, Nahal Oren population of *S. carmeli*) disagree with Gloger's rule, and did exhibit lighter pelage, and this is possibly to blend with lighter soils. Therefore, it is necessary to add the distinction between microgeographic (local) and macrogeographic (regional) scales to Gloger's rule. The northern, mesic but lighter KBZ population indicates that Gloger's rule is secondary to cryptic coloration.

Though Gloger's rule states that the color gradient of dark to light from humid to arid areas fulfills thermoregulatory needs, such a gradient also coincides with concealing coloration, as the soil color is darker in humid and lighter in arid areas. This is because the high density of vegetation is often correlated with high precipitation and humidity. This resulted in the darkening of the earth's surface in humid areas due to moisture and decomposing detritus, whereas arid areas became lighter [42]. Therefore, the selection forces behind concealing coloration and Gloger's rule are alike and obviously inseparable. Taken collectively, pelage coloration of mole rats characterizes both crypsis and Gloger's rule with almost equal exceptions to both.

### Variation in *MC1R* and its implications on mole rats' dorsal coloration

In parallel to the phenotypic difference between northern and southern species, there is also a clear genetic differentiation between the *MC1R* gene variants. The haplotypes that carry valine in position 168 (c.502A>G variant) are exclusively restricted to the lighter southern species, which live in arid environments (*S. carmeli* and *S. judaei*), while the northern darker species, which live in humid environments (*S. galili* and *S. golani*), have methionine in the same position. Although we did not observe a correlation between this *MC1R* genotype and any of the pelage color parameters, there is a strong similarity in the divergence of the MC1R haplotypes distribution between the northern and southern species to the dramatic differences in pheomelanin concentrations in the same groups. This might suggest a functional effect of the MC1R haplotype on pheomelanin, in particular of the non-synonymous variant c.502A>G. However, only molecular biology experiments could prove that this variant is indeed the functional mutation that influences the pelage color. A similar example of MC1R effect was previously shown in a study of subspecies of the Gulf Coast beach mice where a strong association was found between *MC1R* genotypes, pigmentation and background sand brightness, consistent with local adaptation [43]. In pocket mice, four of nine *MC1R* non-synonymous variants were observed in the dark mice from the Pinacate locality. It was suggested that one or more of these four amino acid mutations are responsible for light/dark phenotypic differences seen in the Pinacate population [18]. In our study, the northern populations of mole rats with C-A-C haplotype exhibited higher pheomelanin than southern populations with T-G-C haplotype. Although in the absence of molecular biology studies, we could not confirm that the *MC1R* variants we found, were the causal variants for mole rats' pelage color and had clearly influenced pheomelanin in pelage, our results support that possibility.

The Pheomelanin/eumelanin ratio of *Spalax* populations increased with high latitudes indicating geographical variation. It is likely that higher pheomelanin (together with eumelanin) in northern populations caused darker pelage in *Spalax galili* and *Spalax golani*. We base this claim on report [13] that the darker coat of some rodent species is caused by the deposition of eumelanin and in other species by the deposition of pheomelanin. Geographical variation in melanin contents is known in owls [44], but yet this remains largely untested in rodents despite phenotypic studies often supporting Gloger's rule. However, it is puzzling why eumelanin didn't show such geographic variation in *Spalax*. It appears that eumelanin responds better to soil color variation and pheomelanin responds better to geographical variation in mole rats, but further studies are needed to test whether such a differential function of the two melanins exists and if so, how it optimizes the adaptive roles of dorsal coloration.

### Conclusion

Multiple linear regressions revealed that soil lightness was a determinant of all pelage color coordinates. Darker pelage of mole rats coincides with darker basalt and terra rossa in humid regions, and lighter pelage coincides with lighter rendzina and loess soils in arid regions. Pelage color lightness and eumelanin content of different populations (of all four species) which inhabit the same soil type (in different climatic conditions) converge despite varied soil lightness. Therefore, it is hard to distinguish crypsis from the thermoregulatory function expressed by Gloger's rule. The pelage color differs distinctly between northern and southern species, and the mutations in the *MCIR* gene might therefore be involved. However, it should be noted that other genes in the blind subterranean mole rats genome might also affect the causal chain of its pelage coloration.

## Supporting Information

Table S1 **Populations and species of mole rats screened for Mc1r.** The details of species, populations, soil type and number of animals sampled for each populations was given.
(DOC)

Table S2 **MC1R haplotype frequencies in** ***Spalax*** **species.** The three allelic variants in MC1R gene and the corresponding frequency of occurrence in each species are shown.
(DOC)
