# Correction: Adaptation of Pelage Color and Pigment Variations in Israeli Subterranean Blind Mole Rats, *Spalax Ehrenbergi*

**DOI:** 10.1371/annotation/27bebc65-09c5-4c58-be6c-4f22c4fe0919

**Published:** 2013-08-06

**Authors:** Natarajan Singaravelan, Shmuel Raz, Shay Tzur, Shirli Belifante, Tomas Pavlicek, Avigdor Beiles, Shosuke Ito, Kazumasa Wakamatsu, Eviatar Nevo

The word "ehrenbergi," as part of a species name, was incorrectly capitalized in the title. The correct title is: Adaptation of Pelage Color and Pigment Variations in Israeli Subterranean Blind Mole Rats, Spalax ehrenbergi. The correct citation is: Singaravelan N, Raz S, Tzur S, Belifante S, Pavlicek T, et al. (2013) Adaptation of Pelage Color and Pigment Variations in Israeli Subterranean Blind Mole Rats, Spalax ehrenbergi. PLoS ONE 8(7): e69346. doi:10.1371/journal.pone.0069346. 

