# Supplementary material for: Adaptation of Pelage Color and Pigment Variations in Israeli Subterranean Blind Mole Rats, Spalax Ehrenbergi
Source: PLoS One. 2013 Jul 25;8(7):e69346. doi: 10.1371/journal.pone.0069346 (PMC3723903; doi:10.1371/journal.pone.0069346)
Supplement: Table S1 — Populations and species of mole rats screened for Mc1r. The details of species, populations, soil type and number of animals sampled for each populations was given. (DOC) [file pone.0069346.s001.doc]

Table S1. Populations and species of mole rats screened for Mc1r

| ***N*** | **Soil type** | **Population** | **Species** |
| --- | --- | --- | --- |
| 11 | Rendzina | Kerem-Ben-Zimra | *Spalax galili* (2n=52) |
| 12 | Basalt | Alma | *Spalax galili* (2n=52) |
| 3 | Terra Rossa | Rihaniya | *Spalax galili* (2n=52) |
| 4 | Basalt | Quneitra | Spalax golani (2n=54) |
| 2 | Terra Rossa | Hermon | Spalax golani (2n=54) |
| 6 | Terra Rossa | Muhraka | Spalax carmeli (2n=58) |
| 6 | Terra Rossa | “Evolution Canyon” (Nahal Oren) | Spalax carmeli (2n=58) |
| 13 | Rendzina | Anza | Spalax judaei (2n=60) |
| 11 | Loess | Lahav | Spalax judaei (2n=60) |
