# Supplementary material for: Adaptation of Pelage Color and Pigment Variations in Israeli Subterranean Blind Mole Rats, Spalax Ehrenbergi
Source: PLoS One. 2013 Jul 25;8(7):e69346. doi: 10.1371/journal.pone.0069346 (PMC3723903; doi:10.1371/journal.pone.0069346)
Supplement: Table S2 — MC1R haplotype frequencies in Spalax species. The three allelic variants in MC1R gene and the corresponding frequency of occurrence in each species are shown. (DOC) [file pone.0069346.s002.doc]

Table S2. MC1R haplotype frequencies in *Spalax* species

| **Species** | **C-A-C** | | **C-A-T** | | **T-G-C** | |
| --- | --- | --- | --- | --- | --- | --- |
|  | Number | Percent | Number | Percent | Number | Percent |
| *Spalax galili* | 26 | 100% |  |  |  |  |
| *Spalax golani* | 4 | 67% | 2 | 33% |  |  |
| *Spalax carmeli* | 1 | 8% |  |  | 11 | 92% |
| *Spalax judaei* | 1 | 4% |  |  | 23 | 96% |
